# 

**Published:** 1891-08

**Authors:** 


### CONTENTS:

Article I.—The Physiology and Pathology of the Second Dentition. By Richard Cole Newton, M. D. - - - - 145

" II.—Prophylaxis in the Field of the Dental Surgeon. By Chas. B. Atkinson, D. D. S. - - - - - 163

" III.—Method of Attaching Teeth to Gold Plates by means of Vulcanite. By J. Hall Lewis, D. D. S. - - 169

" IV.—Aneurismal Tumor of the Right Alveolar Process and Vault of the Mouth Treated by Injection. By John S. Marshall, M. D. - - - - - 170

### COMMUNICATIONS.

National Association of Dental Faculties, - - - - 175

National Association of Dental Examiners. - - - - 184

Missouri State Dental Association. - - - - - 188

American Public Health Association. - - - - - 189

The Twenty-fourth Annual Meeting of the American Academy of Dental Science. - - - - - - - - 189

Maryland State Dental Association. - - - - - 190

### EDITORIAL.

Bones Ejected from Her Body. - - - - - - - 190

### MONTHLY SUMMARY.

How to Get a Tin Cast. - - - - - - - - 191

Aristol. - - - - - - - - - 192

